# A Comparison Study on the Therapeutic Effect of High Protein Diets Based on Pork Protein versus Soybean Protein on Obese Mice

**DOI:** 10.3390/foods11091227

**Published:** 2022-04-24

**Authors:** Songsong Jiang, Shanshan Ji, Xinlei Tang, Tao Wang, Hengpeng Wang, Xiangren Meng

**Affiliations:** 1 College of Tourism and Culinary Science, Yangzhou University, Yangzhou 225127, China; 17600189209@163.com (S.J.); jiss1118@163.com (S.J.); tangxinlei0624@163.com (X.T.); wt15950521436@163.com (T.W.); yzuwhp@163.com (H.W.); 2 Key Laboratory of Chinese Cuisine Intangible Cultural Heritage Technology Inheritance, Ministry of Culture and Tourism, Yangzhou 225127, China

**Keywords:** high protein diet, obesity, pork protein, soybean protein, mice

## Abstract

In this study, an obese C57BL/6J mice model was induced to compare the effect of different high protein diets (soybean protein and pork protein) on obesity. The obese mice were randomly divided into four groups: natural recovery (NR), high-fat diet (HF), high soybean protein diet (HSP), and high pork protein diet (HPP) groups. After 12 weeks of dietary intervention, the obesity-related indexes of mice were measured, such as body weight, fat coefficients, blood lipid indexes and so on. Results showed that HSP and HPP decreased the weight and fat coefficients of mice, the levels of serum total cholesterol (TC), low-density lipoprotein cholesterol (LDL-C) and leptin (*p* < 0.05). Soybean protein was shown to be more effective in reducing the weight and fat mass of obese mice, although pork protein seemed to have a better effect on regulating serum triglyceride (TG). In addition, the two high protein diets both alleviated hepatic fat deposition effectively. Furthermore, HPP and HSP decreased the expression of hepatic peroxisome proliferator-activated receptor-γ (PPAR-γ) and increased the protein expression of phosphorylated AMP-activated protein kinase (pAMPK), phosphorylated acetyl CoA carboxylase (pACC), and uncoupling protein 2 (UCP2) (*p* < 0.05). In conclusion, the study shows that high protein diets based on both pork protein and soybean protein alleviated abdominal obesity in mice effectively by regulating lipid metabolism, probably via the UCP2-AMPK-ACC signaling pathway.

## 1. Introduction

Obesity is a chronic metabolic disease characterized by abnormal accumulation of fat in the body due to the imbalance in energy metabolism [1,2]. The prevalence of obesity has increased substantially over the past 40 years, from less than 1% in 1975, to 6–8% in 2016 [3]. According to the World Health Organization, more than 1.9 billion adults are overweight, and more than 650 million adults are obese globally [4]. Approximately 46% of adults and 15% of children in China are obese or overweight [5]. In addition, obesity increases the risk of dyslipidemia and oxidative stress [6,7], which is also closely linked to other common chronic diseases such as cardiovascular disease, type 2 diabetes, hypertension, and certain cancers [8,9]. Moreover, it is thought to be a risk factor for COVID-19 deterioration [10]. So, prevention and treatment of obesity are critical and necessary.

Currently, regulating dietary protein intake is a potential strategy for alleviating obesity and controlling weight [11,12]. As a macronutrient regulation method, high protein diets establish a negative energy balance, by decreasing energy intake and increasing energy consumption, to improve metabolic function [13]. In addition, high protein diets are beneficial in reducing body weight and fat, but it is unclear whether the effect is influenced by protein sources [14,15]. Some studies [16,17] have reported that meat proteins are high-quality protein sources for body growth and health maintenance, with a higher proportion of essential amino acids (EAAs) and better absorption and utilization effects than plant proteins [18,19,20]. Amino acid contents of proteins fluctuate between sources, resulting in diverse impacts on physiology, metabolism, gene, and protein expression [21]. 

In addition, the liver is the primary site of metabolism diseases, such as obesity and diabetes. AMP-activated protein kinase (AMPK) is a key regulator of energy homeostasis, which coordinates metabolic pathways and thus balances energy demand [22]. Peroxisome proliferator-activated receptor-γ (PPAR-γ) is related to lipid metabolism, and its downstream uncoupling protein 2 (UCP2) -AMPK- acetyl CoA carboxylase (ACC) signaling pathway is involved in the regulation of hepatic lipid metabolism [23]. Thus, we speculated that high protein diets could promote the autophosphorylation of AMPK and the phosphorylation of downstream ACC, enhancing fatty acid oxidation and regulating hepatic lipid metabolism. 

Therefore, this study carried out a high protein dietary intervention of different sources on diet-induced obese mice to evaluate the effects of soybean protein and pork protein and explored the associated mechanism. The results will provide appropriate and practical guidance for the dietary intervention of overweight and obese people.

## 2. Materials and Methods

### 2.1. Protein Samples and Animal Diets

Soybean protein isolate (purity 90%) was purchased from Qilu Biotechnology Co., Ltd. (Shandong, China). Pork protein (purity 95%) was from Guohao Biotechnology Co., Ltd. (Xi’an, China).

The feed used in the experiment was granular, including the normal feed, high-fat feed, and two kinds of high protein feed. They were all prepared by Jiangsu synergy Bioengineering Co., Ltd. (Nanjing, China). The high protein feed formula was modified according to the AIN-93G standard formula, and the energy supply ratio of protein was 35%. See Table 1. Composition of different dietary patterns for each feed formula.

### 2.2. Experimental Protocol

A total of 48 4-week-old male C57BL/6J mice (13–16 g) were obtained from Cavens Biogle Model Animal Research Co., Ltd. (Suzhou, China, SCXK (Su) 2018–0002). They were raised in the experimental animal center of Yangzhou University and kept under standard laboratory conditions (temperature (22 ± 1 °C), and humidity (55 ± 5%) with a 12/12 h light/dark cycle (8:00–20:00)). Mice fed and drank freely throughout the experiment.

After a one-week acclimatization period, C57BL/6J mice were randomly divided into the obesity model group (Model group, *n* = 40) and control group (NC group, *n* = 8). They were fed with a high-fat diet or control diet for 16 weeks, respectively. According to the standard [24] that the body weight of the obese mice exceeded 20% of the average body weight of NC group, 32 obese mice were selected and randomly divided into 4 groups: high-fat diet group (HF group, *n* = 8), natural recovery group (NR group, *n* = 8), high soybean protein diet group (HSP group, *n* = 8) and high pork protein diet group (HPP group, *n* = 8). Except for HF, the calories per 100 g of feed of other groups were almost the same. The NC group was fed with control feed for another 12 weeks until the end of the experiment. On week 28, mice were deprived of feed but were given free access to water for 12 h before sacrifice. 

All experimental procedures were approved by the Animal Care and Use Committee of Yangzhou University (No. YXYLL-2021-56) and were performed in accordance with the National Guidelines for the Ethical Review of Experimental Animal Welfare.

### 2.3. Determination of Body Weight and Fat Coefficients

During this experiment, the body weight of mice was measured twice a week. In addition, their mental and physical conditions and feed consumption were monitored every day. After euthanasia, the epididymal fat and perirenal fat of mice were isolated and taken out. The surface tissue fluid was sucked dry with filter paper before weighing, and the ratios of fat mass and body weight were calculated.

### 2.4. Biochemical Analysis

Immediately following euthanasia, blood samples without anticoagulation were collected and centrifuged (3000 rpm) at 4 °C for 10 min to separate the serum. The automatic biochemical analyzer (bs-850, Mindray Inc, Shenzhen, China) was used to measure the levels of total cholesterol (TC), triglyceride (TG), low-density lipoprotein cholesterol (LDL-C), and high-density lipoprotein cholesterol (HDL-C). Leptin and β-Hydroxybutyric acid (β-HB) levels were determined by enzyme-linked immunosorbent assay (ELISA) kits (eBioscience Inc, San Diego, California, USA) according to the instructions. Serum creatinine (SCr) and blood urea nitrogen (BUN) levels were monitored by diagnostic kits (Nanjing Jiancheng Bioengineering Institute, Nanjing, China).

### 2.5. Hematoxylin-Eosin (HE) Staining and Oil Red O (ORO) Staining

When the mice were euthanized, parts of the liver were taken under sterile conditions. The liver tissues of the mice were placed in 4% paraformaldehyde (Servicebio) overnight and dehydrated and embedded in paraffin. Then, liver tissues were sliced into 5-μm-thick sections, fixed on a glass slide, and baked dry. Subsequently, the sections were soaked in xylene (SCRC), ethanol in gradient concentration (SCRC), hematoxylin solution (Servicebio), ethanol in gradient concentration, and eosin dye (Servicebio), respectively, and sealed with neutral gum (10004160, SCRC).

After the fixation of liver tissues, optimal cutting temperature (OCT) compound was added for embedding. Then, the tissues were frozen into lumps and sliced into 5-μm-thick sections. Frozen sections were reheated at room temperature and fixed with 10% paraformaldehyde. After washing, soaking, and dehydration, ORO working solution (Servicebio) was added for staining (10 min) under dark conditions. Subsequently, the sections were sunk in 60% isopropanol (SCRC). Hematoxylin was used for counterstain to visualize the nucleus. Then, the sections were treated with scott tap bluing (Servicebio). Finally, the staining sections were observed under an upright optical microscope (Eclipse E100, Nikon Inc, Shanghai, China) to analyze the histopathological changes, and Image J (National Institutes of Health, Bethesda, MD, USA) was used for morphological semi-quantitative statistical analysis.

### 2.6. Determination of Amino Acid Levels

The amino acid levels of dietary protein were determined by high-performance liquid chromatography. The sample and HCl were added into the hydrolysis tube and put into an oven and hydrolyzed at 110 °C for 22 h. After cooling, it was transferred to a colorimetric tube for constant volume. The sample was taken and put into a centrifuge tube filled with nitrogen, drying at 60 °C for 2 h. Then, 50 μL of derivatization reagent, ethanol:phenylisothiocyanate:water:triethylamine = 7:1:1:1 (prepared just before use, filled with nitrogen) was added. After the 12-week intervention, blood samples with EDTAK2 were used to obtain the plasma. Then, the plasma amino acid level was measured by an Ag1260 high-performance liquid chromatography system (Agilent Technologies Inc, Palo Alto, CA, USA).

### 2.7. RNA Extraction and Quantitative Real-Time Reverse Transcription-Polymerase Chain Reaction (RT-qPCR)

The total RNA in liver samples was extracted with Trizol (15596-026, Invitrogen), and the purity and concentration of RNA were detected with nucleic acid protein quantizer (ds-11, Denovix, Wilmington, Delaware, USA). Reverse transcriptase kits (CW2582M, CWBIO, Beijing, China) were used for reverse transcription of RNA into cDNA in strict accordance with the instructions. After primers, cDNA, and SYBR Green Master Mix were mixed thoroughly, and a real-time PCR instrument (Bio-Rad, Hercules, California, USA) was used to amplify and detect fluorescence intensity with the following parameters: 95 °C for 15 min for 1 cycle; 95 °C for 10 s, 53~58 °C for 30 s, and 72 °C for 30 s for 40 cycles. Gene expression was calculated by the 2^−ΔΔCT^ method, using β-actin as a reference gene. The design of gene primers used for experiments was referred to the primer bank database. In addition, the primer sequences are shown in Table 2. Sequences of primers in RT-qPCR.

### 2.8. Western Blot

When the mice were euthanized, parts of the liver tissue were taken under sterile conditions. The liver tissues were cut into pieces with surgical scissors. After that, they were ground and homogenized in the precooled RIPA protein lysis buffer (50 mM Tris-HCl, pH 7.4, 150 mM NaCl, 1% NP-40, 0.1% SDS) added with protease inhibitor cocktail (Roche, Shanghai, China). The supernatant was taken after centrifugation, which was the extracted total tissue protein. Bicinchoninic acid (BCA) protein quantitative kits (02912e, Cwbiotech, Beijing, China) were used to determine the protein concentration. According to the molecular weight of the target protein, 28 μg protein samples were taken for 10% gel electrophoresis (SDS-PAGE). Briefly, the membranes were transferred by the wet-transfer system, and the PVDF membranes were stained with the ponceau staining reagent. The membranes were blocked in 5% BSA prepared in 1× Tris-buffered saline with 20% Tween 20 (TBST) for 1 h. The membranes were incubated overnight with anti-PPAR-γ (bs-0530R, 1:1000), anti-UCP2 (bs-1926R, 1:1000), anti-pAMPK (bs-4002R, 1:1000), anti-pACC (bs-3039R, 1:1000), anti-AMPK (bs-2771R, 1:1000) or anti-β-actin (bs-10966R, 1:1000) antibody (Bioss, Beijing, China). Then, the membranes were washed with 1× TBST and incubated with HRP-conjugated anti-rabbit (111-035-003, 1:10,000) or anti-goat (115-035-003, 1:10,000) antibody (Jackson) for 40 min at room temperature. Finally, the membranes were incubated in ECL reagents (WBKLS0500, Millipore, Shanghai, China) and scanned using Gel Image System 4.00 (Tanon, Shanghai, China) for semi-quantification analysis.

### 2.9. Statistical Analysis

SPSS 26.0 (IBM, Armonk, NY, USA) was used to analyze all the data. The independent sample t-test was used to compare the data between the two groups; the differences of multiple groups were analyzed by one-way ANOVA and the least significant difference (LSD). *p* < 0.05 was statistically significant, and the results were expressed as mean ± standard deviation (Mean ± SD). GraphPad Prism 5.0 were used for drawing.

## 3. Results

### 3.1. Changes in Body Weight and Physiological Status of Mice

**Figure 1:**
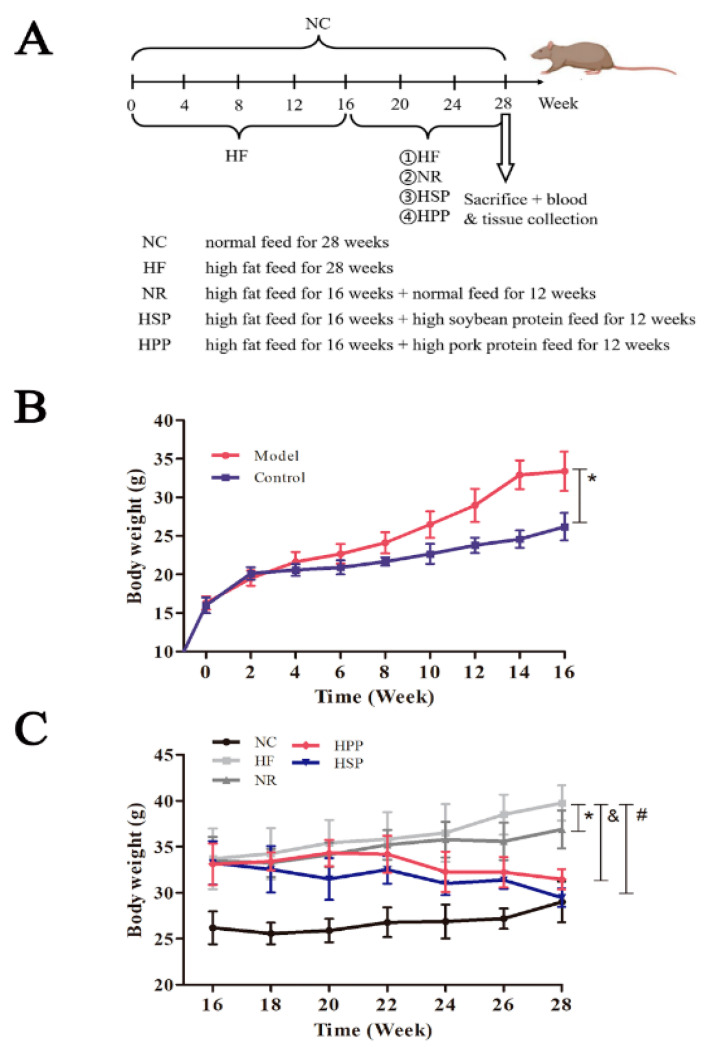
Changes in body weight and physiological status of mice. (**A**) Outline of the animal experimental protocol. C57BL/6J mice were fed with high-fat diets for 16 weeks and then randomly divided into 4 groups for a 12-week intervention. The high-fat diet group (HF group, *n* = 8) was still fed with a high-fat diet, the natural recovery group (NR group, *n* = 8) was fed with a normal diet, the high soybean protein group (HSP group, *n* = 8) was fed with a high soybean protein diet, and the high pork protein group (HPP group, *n* = 8) was fed with a high pork protein diet; the control group (NC group, *n* = 8) was fed with a normal diet for 28 weeks; (**B**) changes in the body weight of Model and Control groups within 16 weeks, * *p* < 0.05, compared with Model and Control groups; (**C**) changes in body weight within the 12-week intervention of five dietary patterns, * *p* < 0.05, compared with NR and HF groups; & *p* < 0.05, compared with HPP and HF groups; # *p* < 0.05, compared with HSP and HF groups.

In this study, diet-induced obese mice were treated with high protein diets (Figure 1A). During the molding, there was no significant variation in food intake between the obese and normal mice. Nonetheless, the mice in the Model group had glossy and oily hair. In addition, the amount of drinking water was increased as compared with the blank control group. After feeding with high-fat diets for 4 weeks, the mice in Model group gained weight significantly compared with the control group (*p* < 0.05). Finally, 32 obese mice were selected to receive a 12-week dietary intervention.

During the intervention, the weight and physiological status of mice in different groups were monitored. The obese mice in NR, HSP, and HPP groups lost weight to varying degrees (Figure 1C). Among them, the weight of mice in the HSP group was significantly decreased compared with the HF group after the 3-week intervention (*p* < 0.05); that of HPP group was significantly decreased compared with HF group after the 7-week intervention (*p* < 0.05); that of NR group was significantly decreased compared with HF group after the 9-week intervention (*p* < 0.05). Furthermore, after 12 weeks, the mice in HSP and HPP groups lost 25.9% and 20.9% more weight than the HF group, respectively. These results indicate that the high protein diets based on soybean protein and pork protein significantly reduced the weight of obese mice, and HSP was more effective.

### 3.2. Effect of Different Proteins on Epididymis and Perirenal Fat Coefficients in Obese Mice

**Figure 2:**
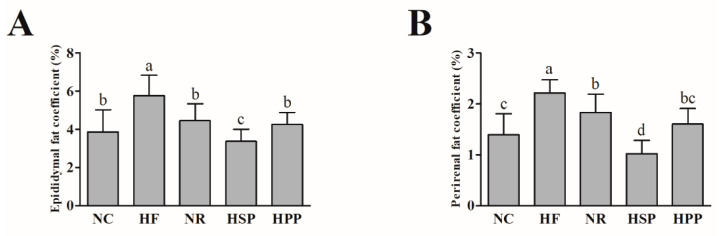
Effect of different proteins on epididymis and perirenal fat coefficients in obese mice. (**A**) Epididymal fat coefficient in each group; (**B**) perirenal fat coefficient in each group. Different letters indicate significant differences between groups (*p* < 0.05).

To further evaluate the effects of high protein diets from different sources on the visceral fat of obese mice, the fat coefficients of mice were measured after the 12-week intervention. As shown in Figure 2, the epididymis and perirenal fat coefficients of NR, HSP, and HPP groups mice were significantly decreased compared with HF group (*p* < 0.05). Notably, there was no statistical difference in epididymal fat coefficients between HPP and NR groups (*p* > 0.05). Moreover, the epididymal and perirenal fat coefficients of HSP group mice were decreased compared with NR and HPP groups (*p* < 0.05), and even NC group (*p* < 0.05), indicating that the high protein diet based on soybean protein showed more benefits in reducing visceral fat in obese mice than pork protein.

### 3.3. Effect of Different Proteins on Serum TC, TG, LDL-C, and HDL-C in Obese Mice

**Figure 3:**
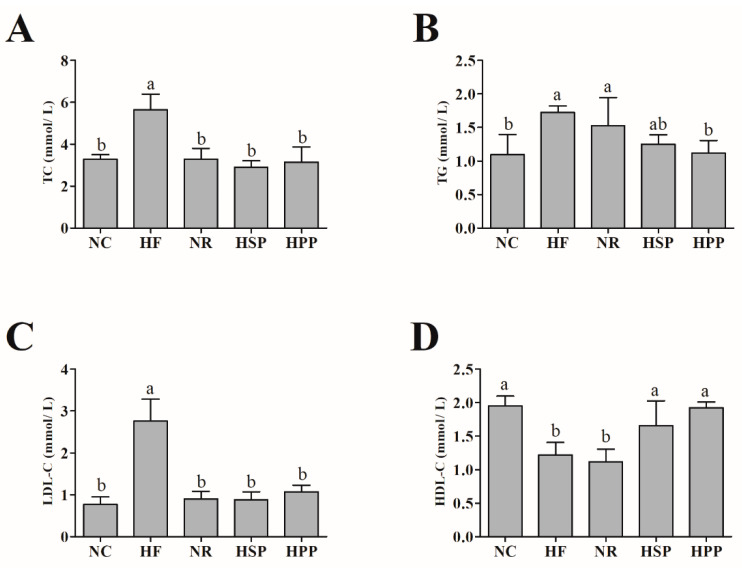
Effect of different proteins on serum lipid indexes in obese mice. (**A**) Serum total cholesterol (TC) level of mice in each group; (**B**) serum triglyceride (TG) level of mice in each group; (**C**) serum low-density lipoprotein cholesterol (LDL-C) level of mice in each group; (**D**) serum high-density lipoprotein cholesterol (HDL-C) level of mice in each group. Different letters indicate significant differences between groups (*p* < 0.05).

Blood lipids usually reflect the state of lipid metabolism [25,26]. Therefore, the levels of serum TC, TG, LDL-C, and HDL-C of mice were measured. As shown in Figure 3, compared with the HF group, the serum TC and LDL-C levels of obese mice in the NR, HSP, and HPP groups were significantly reduced (*p* < 0.05). Interestingly, only HPP reduced the serum TG level of obese mice (*p* < 0.05). The serum TG level of mice in the HSP group was slightly decreased compared with HF group, but there was no statistical significance (*p* = 0.051). In addition, HSP and HPP groups significantly increased the level of serum HDL-C compared with HF and NR groups (*p* < 0.05). From what has been discussed above, the different sources of high protein diets both regulated the blood lipid levels of obese mice.

### 3.4. Effect of Different Proteins on Serum Leptin and β-HB in Obese Mice

**Figure 4:**
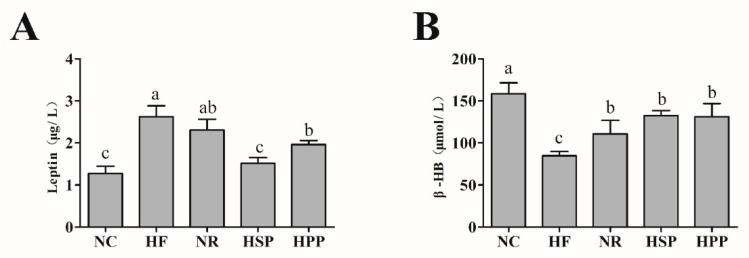
Effect of different proteins on serum metabolic indexes in obese mice. (**A**) Serum leptin level of mice in each group; (**B**) serum β-hydroxybutyrate (β-HB) level of mice in each group. Different letters indicate significant differences between groups (*p* < 0.05).

Leptin inhibits appetite and enhances heat production, which is strongly associated with body fat [27]. To investigate the impact of different proteins on fat deposition and metabolic capability, serum leptin and β-HB levels were measured. As shown in Figure 4, the serum leptin levels of HSP and HPP group mice were decreased compared with the HF group (*p* < 0.05). In comparison with the HPP group, the leptin level of HSP group mice was also decreased significantly (*p* < 0.05), which was positively correlated with the decrease of visceral fat mass. Moreover, in terms of ketone, the serum β-HB levels of NR, HSP, and HPP groups were increased significantly (*p* < 0.05). These results demonstrate that high protein diets successfully modulated blood leptin levels in obese mice. In addition, the intervention of high protein diets and regular diets accelerated the metabolism of fatty acids in the liver of obese mice, to promote the production of β-HB in the body.

### 3.5. Effect of Different Proteins on SCr and BUN in Obese Mice

**Figure 5:**
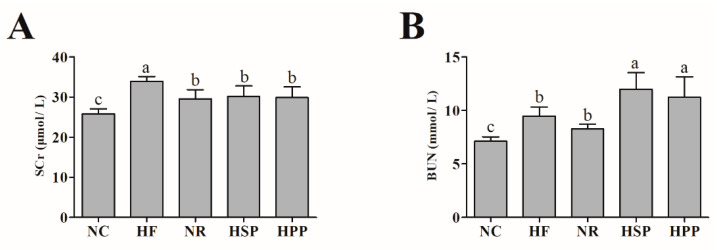
Effect of different proteins on kidney functions in obese mice. (**A**) Serum creatinine (SCr) level of mice in each group; (**B**) blood urea nitrogen (BUN) level of mice in each group. Different letters indicate significant differences between groups (*p* < 0.05).

To explore whether high protein diets have side effects on the kidneys of obese mice, the SCr and BUN levels were measured in each group. As shown in Figure 5, compared with the NC group, the levels of SCr in other groups were significantly increased (*p* < 0.05). It is worth noting that the SCr levels of HPP and HSP groups were decreased in comparison with the HF group. HSP and HPP increased the BUN level in obese mice (*p* < 0.05), and this may be caused by excessive amino acid metabolism. These results indicate that the high fat diet for a long period of time had certain side effects on the kidneys, while the two high protein diets did not in this study.

### 3.6. Effect of Different Proteins on Liver and Fat Histomorphology in Obese Mice

**Figure 6:**
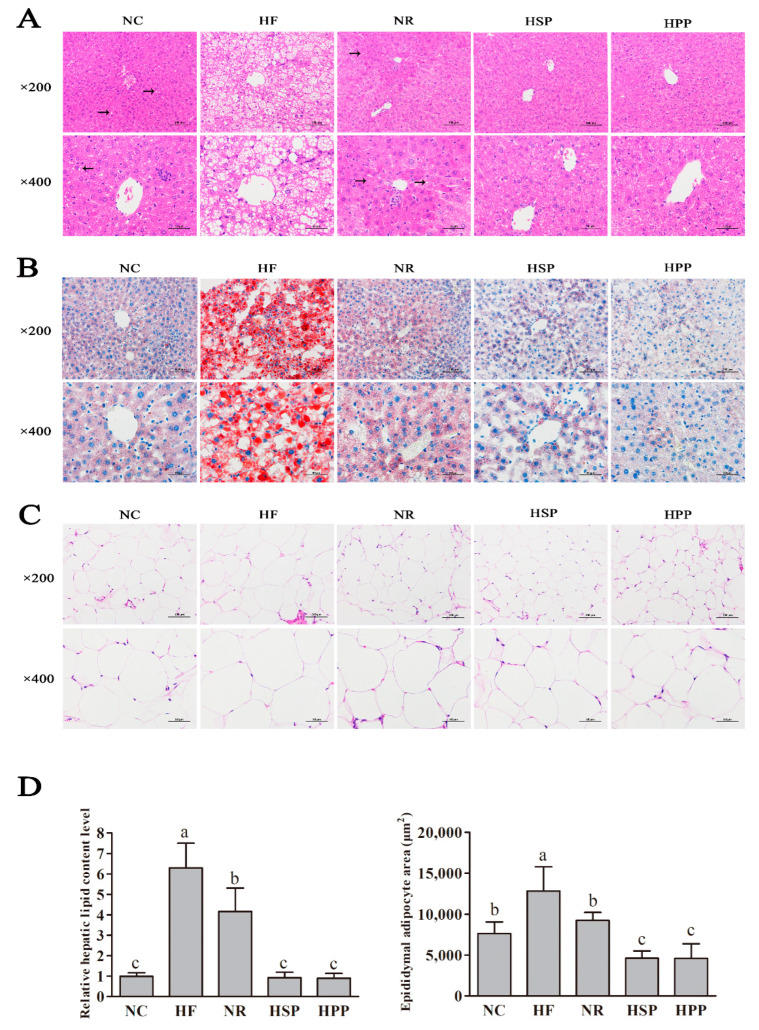
Effect of different proteins on liver and fat histomorphology in obese mice. (**A**) Hematoxylin-eosin (HE) staining results of liver sections of mice in each group; (**B**) Oil red O (ORO) staining results of liver sections of mice in each group; (**C**) HE staining results of epididymal fat sections; (**D**) semi-quantification of ORO and HE staining by Image J. Different letters indicate significant differences between groups (*p* < 0.05).

Diet-induced obesity causes morphological changes of liver tissues and steatosis of hepatocytes [28,29,30]. As shown in Figure 6, the HE staining of the liver showed that HF group mice had much steatosis in hepatocytes, the unclear boundary of hepatic lobules, the disordered arrangement of the hepatic cord, and a large number of lipid droplets and vacuoles. However, no obvious vacuoles were observed in the hepatocytes of HSP and HPP groups. In parallel, the ORO staining of the liver also suggested similar results. There were a large number of red lipid droplets in the liver sections of mice in the HF group and almost no lipid droplets in the liver sections of mice in the HSP and HPP groups. 

In addition, the HE staining of epididymal fat sections showed that the epididymal adipocytes in the HF group became larger significantly, and those in the HSP and HPP groups became smaller significantly compared with the NR group. Image J was used for morphological semi-quantitative statistical analysis. Compared with the HF and NR groups, the HSP and HPP groups decreased the relative hepatic lipid level (*p* < 0.05). In addition, the average area of epididymal adipocytes in the NR, HSP, and HPP groups was decreased significantly compared with the HF group (*p* < 0.05). Meanwhile, compared with the NR and NC groups, the area of epididymal adipocytes in the HSP and HPP groups was also significantly decreased (*p* < 0.05). These results indicate that the high protein diets based on pork protein and soybean protein effectively reduced the lipid degeneration of the liver and visceral fat deposition in obese mice.

### 3.7. Dietary Amino Acid (AA) Components of Different Proteins and Effect on Plasma Amino Acids in Obese Mice

**Figure 7:**
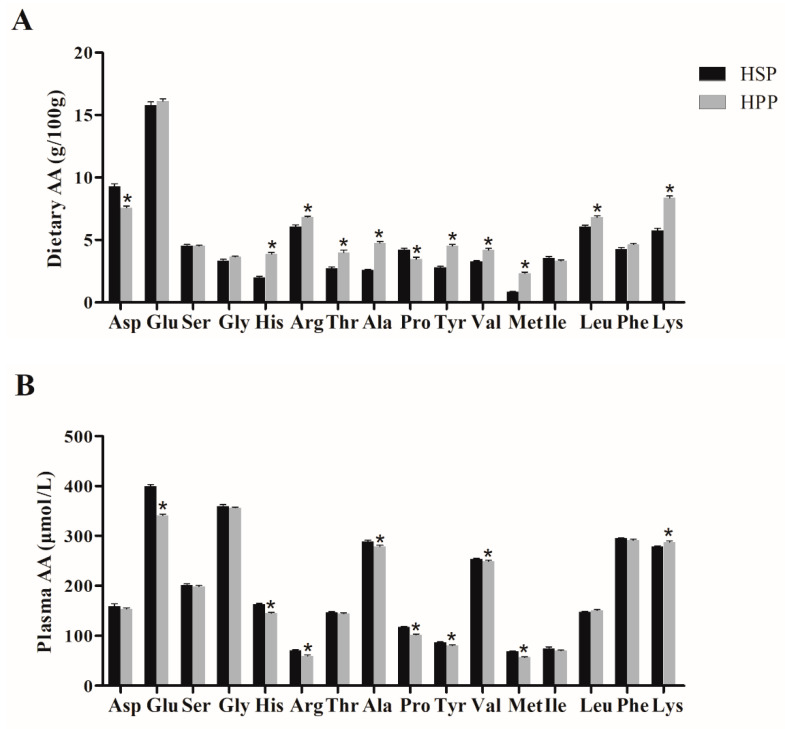
Dietary amino acid (AA) components of different proteins and effect on plasma amino acids in obese mice. (**A**) Dietary amino acid components of soybean protein and pork protein; (**B**) effect of diets on plasma amino acid levels. * *p* < 0.05, compared with HSP group.

To explore the mechanism of different effects of soybean protein and pork protein on lipid metabolism in obese mice, the dietary amino acid components of soybean and pork proteins and the plasma amino acid components of HSP and HPP group mice were measured. As shown in Figure 7, the levels of most dietary amino acids of pork protein were significantly higher than those of soybean protein, such as methionine, valine, alanine, tyrosine, leucine, and arginine. Meanwhile, the levels of total amino acids (TAAs), branched-chain amino acids (BCAAs), and essential amino acids (EAAs) of pork protein were higher. Soybean protein demonstrates methionine restriction. However, after 12-week dietary intervention, most of the plasma amino acids levels of mice in the HPP group were the same as or at even lower levels than those in the HSP group, indicating that the amino acid advantage of pork protein was wiped out by consumption, absorption, and metabolism.

### 3.8. Effect of Different Protein Sources on the Expression of Hepatic PPAR-γ, UCP2, pAMPK, and pACC in Obese Mice

**Figure 8:**
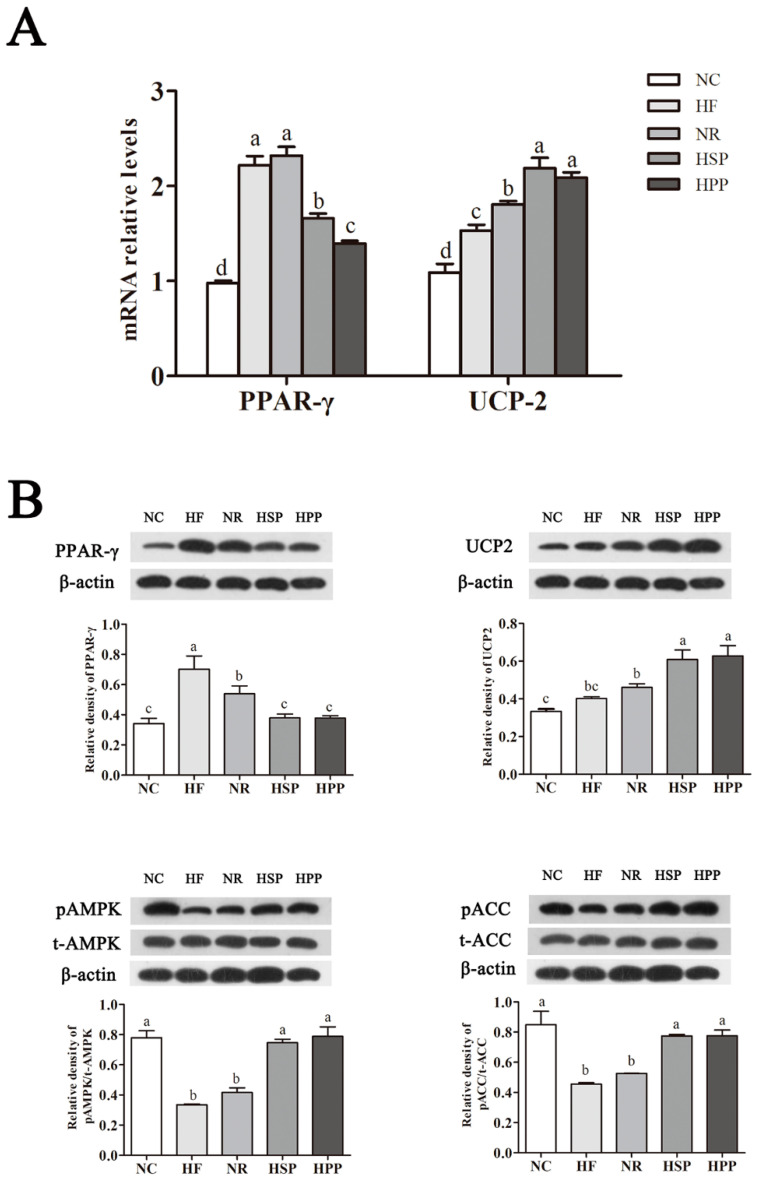
Effect of different protein sources on the expression of hepatic peroxisome proliferator-activated receptor-γ (PPAR-γ), uncoupling protein 2 (UCP2), phosphorylated AMP-activated protein kinase (pAMPK), and phosphorylated acetyl CoA carboxylase (pACC) in obese mice. (**A**) Effect of different proteins on hepatic PPAR-γ and UCP2 on gene expression in obese mice; (**B**) effect of different proteins on hepatic PPAR-γ, UCP2, pAMPK, and pACC on protein expression in obese mice. Different letters indicate significant differences between groups (*p* < 0.05).

PPAR-γ and its downstream molecules have shown to be beneficial to lipid metabolism [23]. To further study the mechanism of different sources of high protein diets affecting hepatic lipid metabolism, we explored the effect of different dietary modes on lipid metabolism-related gene and protein expression in the liver of obese mice. As shown in Figure 8A, compared with HF and NR groups, the mRNA expression of hepatic PPAR-γ in HSP and HPP group mice was decreased significantly (*p* < 0.05); the mRNA expression of hepatic PPAR-γ in HPP group mice was decreased compared with HSP group (*p* < 0.05). At the same time, the mRNA expression of hepatic UCP2 in the HSP and HPP groups was significantly increased compared with the other groups (*p* < 0.05); there was no significant difference between the two groups (*p* > 0.05).

In addition, compared with NC group, the protein expression of hepatic PPAR-γ in the HF group was significantly increased (*p* < 0.05); the protein expression of hepatic PPAR-γ in mice in the HSP and HPP groups was significantly decreased compared with the NR group (*p* < 0.05). In addition, HSP and HPP significantly increased the protein expression of the hepatic UCP2 of mice (*p* < 0.05). Furthermore, the protein expression of hepatic pAMPK (to t-AMPK) of the HSP and HPP groups was significantly increased compared with the HF and NR groups (*p* < 0.05), and there was no significant difference between the NR and HF groups (*p* > 0.05). Compared with the HF and NR groups, the protein expression of the hepatic pACC (to t-ACC) of the HSP and HPP groups was significantly increased (*p* < 0.05). As a result, high protein diets promoted the expression of hepatic UCP2 in obese mice, reduced the expression of PPAR-γ, and accelerated the oxidative decomposition of fat in the liver.

## 4. Discussion

Different dietary proteins often play distinctive roles in the human body [31,32,33,34]. The objective of this study is to investigate the different effects of pork and soybean proteins on obese mice to explore this dietary therapy for obese people. In this experiment, we compared the weight and physiological state of obese mice, visceral fat coefficients, and other relevant indicators to evaluate the therapeutic effect of different high protein diets on obese mice. Furthermore, the possible mechanisms were preliminarily explored.

Although some animal studies have identified the different effects of plant and meat proteins on changing lipid, energy and amino acid metabolism, as well as the insulin signaling pathway [33], there have been few studies on the specific roles of different protein sources in changing lipid metabolism in certain disease states. Our findings revealed that after the long-term intervention, high protein diets based on soybean protein and pork protein reduced the weight and adipose tissues of already obese mice to varying degrees. During the 12-week dietary intervention, there was no significant difference in the food intake of the mice in different groups, which suggested that the weight loss had nothing to do with the food intake. From the comparison of the fat coefficients among groups, it was speculated that the weight loss of obese mice could be attributed to the fall in fat mass rather than lean weight. Leptin is a hormone released by adipose tissues in direct proportion to their mass, and it can enhance energy release [35]. Therefore, we considered that the decrease of serum leptin levels in the HSP and HPP groups was closely correlated to the loss of adipose tissues. Simultaneously, the levels of pAMPK and pACC protein expression in the liver and serum β-HB were increased, indicating that high amounts of soybean protein and pork protein intake could convert excess amino acids and fats into ketones through indirect oxidization [36]. At the same time, the levels of SCr and BUN indicated that the 12-week high protein diets did not cause additional renal impairment in obese mice. The staining results of liver and fat sections suggested that long-term high protein diets reduced fat accumulation in the liver of abdominal obese mice, relieved hepatic steatosis, and remolded fatty liver. Furthermore, we observed that soybean protein in reducing the mass of visceral fat was more effective than pork protein, which was similar to the findings of Liisberg [37] and Myrmel [38]. They found that the high-fat and high-protein (HP/HF) diets from different protein sources showed great differences in the capacity of regulating the development of obesity in lean mice. Only the diet based on casein successfully prevented obesity induced by high-fat diets, while the diets based on pork and chicken proteins were even more likely to lead to obesity. At the same time, pork protein diets decreased the expression of uncoupling protein 1 (UCP1), deiodinase iodothyronine type II (DIO2), and peroxisome proliferator-activated receptor-gamma coactivator-1α (PGC-1α), resulting in the gain of body weight and fat mass in mice [37]. This suggested that the role of dietary protein sources in high protein diets was more important in preventing obesity than reversing it. Conversely, Shi et al. [39] found that pork protein reduced the mass of epididymal fat and liver in rats compared with soybean protein and other dietary proteins. In our study, high protein diets based on pork protein and soybean protein effectively decreased the weight and visceral fat coefficients of obese mice after the 12-week intervention. These different results might be related to the animal strains, protocols, and so on.

In addition, we found that long-term HPP and HSP decreased the serum TC concentration of obese mice; HPP reduced the serum TG concentration, whereas HSP had no obvious effect. Moreover, both patterns of the two dietary proteins decreased the levels of serum LDL-C and increased the levels of serum HDL-C. These findings were consistent with previous results. Brandsch [40] found that pork protein decreased the concentration of plasma TG in rats compared with casein, and the concentration of plasma TG in rats was increased due to the intake of soybean protein. In parallel, several previous studies showed that the highly expressed scavenger receptor class B type I (SR-BI) gene in the liver of rats reversed cholesterol absorption and decreased cholesterol levels via comparatively high bile acid synthesis after ingesting pork protein [39,41]. Compared with casein, soybean protein inhibited cholesterol absorption and boosted cholesterol degradation to generate bile acids [42]. As a result, it was suspected that the two dietary proteins activated the expression of the low-density lipoprotein receptor (LDLR) gene via the cholesterol negative feedback regulatory mechanism and promoted the liver to absorb cholesterol from LDL.

To explore the mechanisms of the different effects of soybean protein and pork protein in the treatment of obesity, the amino acid levels in dietary proteins and plasma were analyzed and compared. The amino acid composition of dietary protein can affect its metabolism in the body [43]. It is worth noting that soybean protein resulted in methionine restriction. The content of dietary methionine was strongly positively correlated with the serum cholesterol level. Dietary methionine restriction enhances the expression of fibroblast growth factor 21 (FGF21) and activates the AMPK/PGC-1α signal to regulate lipid metabolism [44,45,46]. However, we found that the levels of BCAAs and aromatic amino acids (AAAS) in the pork protein were higher than those in soybean protein. BCAAs have proven to effectively improve lipid metabolism and reduce the concentration of TG in the liver and skeletal muscle [47]. Under this, the higher contents of BCAAs in pork protein explained, in part, the better ability to reduce blood lipid. In addition, the content of leucine in pork protein was also higher than soybean protein; leucine is a ketogenic amino acid that can reduce fat production by inducing autophagy-related molecules [48]. Moreover, the decrease of plasma amino acid levels after ingesting pork protein might be due to the insufficient absorption of amino acids by the small intestine, or the sufficient metabolism of amino acids of mice in the HPP group. Proteins from various sources influenced the body’s amino acid supply through their amino acid components, as well as the body’s protein synthesis via the Mammalian Target of Rapamycin (mTOR) signal pathway, which senses amino acid abundance. When sufficient dietary amino acids are consumed, p70 ribosomal protein S6 kinase (p70S6K) is activated through the mTOR signal pathway to phosphorylate eukaryotic translation initiation factor 4E binding protein 1 (4EBP1), which promotes protein translation and expression; nevertheless, amino acid deficiency causes the opposite condition [49].

Overexpression of PPAR-γ in the liver led to the accumulation of fat in liver tissues [50,51]. At the recommended protein levels, the hepatic PPAR-γ transcription levels of growing rats fed with pork protein were decreased compared with that of rats fed soybean protein [41], which is similar to our research results. We also found the lower gene expression of the hepatic PPAR-γ of mice in the HPP group. Nonetheless, from the protein expression, the expression levels of PPAR-γ in the liver of already obese mice had no statistical difference (*p* > 0.05). As a proton carrier, uncoupling protein (UCP) on the inner membrane of mitochondria changed the electrochemical gradients on both sides of the membrane by transporting H^+^ and uncoupled the processes of oxidation and phosphorylation [52]. These changes resulted in the decrease of ATP generation and the increase in energy consumption. The enzymes in the mitochondrial electron transport chain of the rat liver after long-term intake of meat protein were mainly mildly expressed at the protein level, especially ATP synthase [53]. From this, there was an uncoupling phenomenon between oxidation and phosphorylation in mice with the intake of meat proteins, promoting adaptive heat production. In addition, UCP2 was widely distributed in most mammalian tissues and activated AMPK [23,54]. It promoted the phosphorylation of downstream ACC through the increase of autophosphorylation, to enhance the oxidation of fatty acids and regulate lipid metabolism [55,56]. The AMPK-ACC pathway was shown to regulate lipid metabolism in obese rats [57,58], and PPAR-γ is a known regulator of UCP2 [59]. In this study, the two dietary proteins reduced the expression of hepatic PPAR-γ in obese mice and enhanced the expression of UCP2. Meanwhile, the protein expression of pAMPK and pACC were increased. According to the latest research results, high protein diets from different sources aided in weight loss by regulating intestinal flora to participate in energy and lipid metabolism [60,61]. Thus, the regulation of pork protein and soybean protein on intestinal flora is hypothesized to be the reason for their different therapeutic effects on obesity; this needs further study. 

## 5. Conclusions

To summarize, the study demonstrated that high protein diets based on both pork protein and soybean protein effectively alleviated nutritional obesity in already obese mice, which might regulate hepatic lipid metabolism via the UCP2-AMPK-ACC signaling pathway. In terms of apparent indicators, there were differences in the therapeutic effects of pork protein and soybean protein on obesity in mice. Soybean protein was shown to be more effective in reducing weight and the fat mass of obese mice, although pork protein seemed to have a better effect on regulating serum TG. These discrepancies might be due to the amino acid composition and protein metabolic properties. It is worth noting that, considering the species differences between humans and animals as well as the dietary complexity, the results of this study need further verification in human beings. Despite this, we think that these findings will provide scientific strategies and suggestions for better weight management.

## Figures and Tables

**Table 1:** Composition of different dietary patterns.

	Groups									
	NC		HF		NR		HSP		HPP	
Ingredient	gm	kcal	gm	kcal	gm	kcal	gm	kcal	gm	kcal
Pork protein	0	0	0	0	0	0	0	0	368.42	1400
Soy protein	0	0	0	0	0	0	388.89	1400	0	0
Casein	200	800	200	800	200	800	0	0	0	0
L-cystine	3	12	3	12	3	12	3	12	3	12
Corn starch	506.2	2024.8	0	0	506.2	2024.8	304.61	1218.44	325.08	1300.32
Maltodextrin	125	500	125	500	125	500	116	464	116	464
Sucrose	68.8	275.2	68.8	275	68.8	275.2	50	200	50	200
Cellulose	50	0	50	0	50	0	50	0	50	0
Soybean oil	25	225	25	225	25	225	40	360	40	360
Lard	20	180	245	2205	20	180	0	0	0	0
Vitamin mix V10037	10	40	10	40	10	40	10	40	10	40
Mineral mix S10022G	35	0	35	0	35	0	35	0	35	0
Line bitartrate	2.5	0	2.5	0	2.5	0	2.5	0	2.5	0
Total	1045.5	4057	764.3	4057	1045.5	4057	1000	3694.44	1000	3776.32

NC: the control group; HF: the high-fat diet group; NR: the natural recovery group; HSP: the high soybean protein diet group; HPP: the high pork protein diet group.

**Table 2:** Sequences of primers in RT-qPCR.

Gene	Upstream Primer (5′-3′)	Downstream Primer (3′-5′)
PPAR-γ	TCGCTGATGCACTGCCTATG	GAGAGGTCCACAGAGCTGATT
UCP2	ATGGTTGGTTTCAAGGCCACA	CGGTATCCAGAGGGAAAGTGAT
β-actin	GGCTGTATTCCCCTCCATCG	CCAGTTGGTAACAATGCCATGT

## Data Availability

Data is contained within the article.
